# 334. Clinical Outcomes with Implementation of Accelerate Pheno Blood Culture Detection System for Gram Negative Bacteremia

**DOI:** 10.1093/ofid/ofac492.412

**Published:** 2022-12-15

**Authors:** Shu Xian Lee, Benita Y Wu, Kurt Suter, Matthew S Lokant, Andrew Ward, Amy E Spigelmyer, Lauren K Freeman, Jesse M Thompson, Ryan Demkowicz, Catessa A Howard, Rocco LaSala, Rebecca Reece

**Affiliations:** West Virginia University, Morgantown, West Virginia; West Virginia University, Morgantown, West Virginia; West Virginia University, Morgantown, West Virginia; West Virginia University, Morgantown, West Virginia; West Virginia University, Morgantown, West Virginia; West Virginia University, Morgantown, West Virginia; WVU Medicine, Morgantown, West Virginia; West Virginia University, Morgantown, West Virginia; West Virginia University, Morgantown, West Virginia; West Virginia University, Morgantown, West Virginia; West Virginia University, Morgantown, West Virginia; West Virginia University, Morgantown, West Virginia

## Abstract

**Background:**

Bacteremia is a life-threatening illness. Delayed treatment increases patient morbidity and healthcare costs. Accelerate Pheno™ Blood Culture Detection System (AXDX) is a novel diagnostic technology for rapid detection of gram-negative bacteremia. Studies have shown accurate and faster time to speciation and sensitivity (TTSS) by AXDX compared to conventional modality. Our unique study examined the direct impact of AXDX on clinical outcomes and cost.

**Methods:**

This retrospective study consisted of 213 patients aged 18 years and older admitted to our academic institution with gram-negative bacteremia. The pre-AXDX group had 109 patients admitted in 2019 and the post-AXDX group had 104 patients admitted in 2021. Demographics, microbes, TTSS, time to de-escalation of therapy (TTDeT), length of stay (LOS), readmissions, mortality rates, and *Clostridioides difficile* infection (CDI) rates were recorded.

**Results:**

The pre-AXDX group had 51.4% females, mean age of 60.3 years, mean Charlson Comorbidity Index (CCMI) of 2.2, mean LOS of 21.2 days, and mean Pitt Bacteremia Score (PBS) of 2.4. The post-AXDX group had 52.0% females, mean age of 63.7 years, mean CCMI of 3.0, mean LOS of 15.0 days, and mean PBS of 2.7. Both groups’ top 2 sources of bacteremia were urinary and gastrointestinal and top 2 microbes were *Escherichia coli* and *Klebsiella pneumoniae*. Pre-AXDX's mean TTSS was 71.9 hours and 23.6 hours for post-AXDX. Pre-AXDX's mean TTDeT was 74.0 hours and 43.9 hours for post-AXDX. The pre-AXDX cohort had 7.4% more related readmissions, 5.5% more CDI, and 0.3% more inpatient mortality than post-AXDX group.

**Conclusion:**

In addition to faster TTSS with AXDX as seen with previous studies, our study shows clinical advantages with AXDX use. Both groups were comparable in bacteremia sources and microbes. The post-AXDX group had higher CCMI and PBS scores, indicating they were more ill. Despite this, the pre-AXDX group had 30.05 hours longer TTDeT, 6.17 days longer mean LOS, 5.45% more CDI, 7.12% more readmissions, and 0.26% higher mortality. The pre-AXDX group also reported adverse reactions to antibiotics, while post-AXDX did not. Our data shows AXDX use improves clinical outcomes with reduced adverse effects, mortality and CDI rate and lower costs with less LOS and readmission rates.

**Disclosures:**

**All Authors**: No reported disclosures.

